# Nutritional status significantly affects hospital length of stay among surgical patients in public hospitals of Northern Ethiopia: single cohort study

**DOI:** 10.1186/s13104-019-4451-5

**Published:** 2019-07-15

**Authors:** Mulugeta Woldu Abrha, Oumer Seid, Kidanu Gebremariam, Amha Kahsay, Haftom Gebrehiwot Weldearegay

**Affiliations:** 1 Tigray Health Research Institute, Mekelle, Ethiopia; 2 School of Public Health, College of Health Sciences, Mekelle University, Mekelle, Ethiopia; 3 Department of Midwifery, College of Health Sciences, Mekelle University, Mekelle, Ethiopia

**Keywords:** Nutritional status, Length of hospital stay, Duration, Surgical patients, Northern ethiopia

## Abstract

**Objective:**

This study aimed to assess the effect of nutritional status on length of hospital stay in Northern Ethiopia.

**Result:**

Institutional based prospective cohort study was conducted among 324 admitted surgical patients. Length of hospital stay were significantly associated with duration of disease (Adjusted Hazard Ratio (AHR) = 3.7,95% Confidence interval (CI):2.35–5.82), history of surgery (AHR = 1.4, 95% CI 1.40, 95% CI 1.17–1.86), nutritional status (Body Mass Index (AHR = 1.38, 95% CI 1.28–1.51), Mid Upper Arm Circumference (AHR = 1.29, 95% CI 1.04–1.62)) and individual diet diversity score (AHR = 2.64, 95% CI 1.14–6.14). Screening of patients for malnutrition at admission and provision of dietary supplements based on their nutritional status is recommended.

**Electronic supplementary material:**

The online version of this article (10.1186/s13104-019-4451-5) contains supplementary material, which is available to authorized users.

## Introduction

Length of hospital stay (LOS) is one of the key performance indicators for hospital management and efficiency of the health system. Reducing hospital stay has the potential to decrease health care cost, risk of infections, other hospital acquired diseases and improves patients' quality of life [1-3]. Under nutrition increases length of hospital stay due to reduced wound healing, increased complication rates, mortality, and healthcare costs. Previous studies found that up to 40% of patients are malnourished at the time of admission and majority of these patients continued to be nutritionally depleted throughout their hospital course [4, 5].

Malnourished surgical patients have complication and mortality rates 3 to 4 times higher than normally nourished patients with longer hospital admissions, incurring up to 50% greater costs. It is often difficult to separate the deleterious effects of malnutrition from the underlying disease process itself, especially because each can be a cause or consequence of the other [5-9].

The cost–benefit associated with nutritional intervention in patients at risk of malnutrition found to be estimated saving to the health care facility of USD1.064 per patient [10]. Despite numerous advances in medicine and clinical care, the simple correction of a patient’s nutritional status are not considered as a sufficient medical priority [11].

Even though there are studies from developed countries, there are limited studies concerning nutritional status and hospital length of stay among surgical patients in Ethiopia. Therefore this study examined nutritional status and factors associated with length of hospital stay among admitted surgical patients and might have an input to the health care professionals and policy makers.

## Main text

### Methods

#### Study setting, design and sample size

Institutional based prospective cohort study design was conducted in Tigray Region, Northern Ethiopia from January–March 2016. The source population was all admitted surgical patients older than 18 years of age in hospitals. Surgical patients were patients admitted to the surgical ward for surgical intervention. The sample size was calculated using double proportion formula taking malnutrition as exposure variable [6]. The desired level of precision was set at 0.05 and considering the effect of multi-stage sampling; a design effect of 1.5 was used. Adding 10% of possible non-responses, total sample size was 324 patients. Tigray Region has seven zones and out of these two zones was selected randomly. There were five general hospitals in the selected two zones. Then after, three hospitals were selected by lottery method. Likewise, study participants were proportionally allocated to the selected hospitals based on their amount of bed. Finally the participants were consecutively included to the study during admission to surgical ward.

#### Data collection procedure and quality improvement

Data were collected using standardized, structured and face to face interviewer questionnaire and direct measurement. Questionnaire had socio-demographic information, clinical variables, and dietary assessment and biochemical tests sections. Data quality was managed by trained three Bachelors of Science holders fluent in the local language (Tigrigna). A modification was made after a pre-test. Daily supervision, spot checking and reviewing completed questionnaire was conducted. Duplicate measurements of weight, height, mid upper arm circumference and waist circumference was taken at the same time from each study subject during admission using calibrated and standardized equipment and average value of the measurement was recorded.

#### Anthropometric measurements

*Weight* determined by a scale with a maximum capacity of 150 kg and accuracy of 0.1 kg and was calibrated after each measurement.

*Height* measured in standing position in stadiometer but for those confounded to bed were used demi-span and arm span based on the patient’s condition.

*Body mass index* calculated dividing weight by height square and classified as underweight (BMI < 18.5 kg/m^2^), normal weight (18.5–24.9 kg/m^2^), overweight (25–29.9 kg/m^2^), Obese (BMI ≥ 30 kg/m^2^).

*Waist circumference (WC)* was measured on the median line of costal border and the iliac crest at the end of exhalation. Dichotomize into higher than 94 cm for men and 80 cm for women which were considered at risk according to IDF [12].

*MUAC* measured using a non-stretchable MUAC tape and was dichotomize for male < 20 cm and male < 26 male considered as under nutrition and ≥ 20 cm (female), ≥ 26 cm (male) considered as well nourished.

*Hemoglobin status* was collected from the medical records and cut-off point was 11 g/dl [12]. *Dietary assessment* assessed to patients what they eat with in the past 24 h and were categorized ≤ 3 as low IDDS, 4–5 as medium IDDS and ≥ 6 as high IDDS**.**

#### Statistical analysis

Data was entered in to Epi Data 3.1 statistical software and analyzed using SPSS version 20.0 package. Descriptive statistics were computed after normality checked. Patient outcomes other than discharge (died, defaulter, patients self-discharge and patients referred to other hospital) were considered as censored. Finally, the outcome of each subject was dichotomized into censored or discharged. The relationship between length of hospital stay and the covariates was analyzed using Cox bi-variable proportional regression model and before fitting the covariate into the model proportional hazard assumption was checked by examining Log (-Log) S (t) plots. In order to identify independent predictors a multivariable Cox-proportional adjusted model was fitted with those variables p-value ≤ 0.25 in bi-variate Cox proportional regression analysis. Crude and adjusted hazard ratios with their 95% Confidence interval (CI) were estimated and P-value less than 0.05 were used to declare the presence of significant association.

### Result

#### Socio-economic and demographic characteristics of study participants

The study involved 324 patients above 18 years old. The mean (SD) age of the participant were 39 ( ± 15) and three fifth 210 (62%) of them were in the age range of 18–40 years. Slightly above half 171 (52.8%) of them were rural residents. Male accounts two third 210 (64.8%) of the study subject. Six out of ten 211 (65.1%) of the respondents had 3–5 family members. Two fifth 137 (42.3%) of the participants had no formal education. Six out of ten (n = 212) of the patients were married. Half of the participants used public car 175 (54%) when they are transported to Hospitals. One third of participants 119 (36.7%) were farmers (Table1).

**Table 1:** Socio-economic and demographic characteristics of surgical patients Northern Ethiopia, 2016 (N = 324)

Characteristics	Frequency	Percent
Age in year		
18–40	201	62.0
41–64	97	29.9
> 64	26	8.0
Sex		
Female	114	35.2
Male	210	64.8
Religion		
Orthodox	282	87.0
Muslim	42	13.0
Residence		
Rural	171	52.8
Urban	153	47.2
Educational status		
No formal education	137	42.3
Grade 1–4	22	6.8
Grade 5–8	35	10.8
Grade 9–10	49	15.1
Grade 11–12	29	9.0
College and above	52	16.0
Marital status		
Married	212	65.4
Divorced	26	8.0
Widowed	20	6.2
Single	66	20.4
Family members		
1–2	42	13.0
3–5	211	65.1
6–7	63	19.4
> 7	8	2.5
Transportation type to hospital		
Foot	31	9.6
Private care	5	1.5
Public care	175	54.0
Government car	12	3.7
Ambulance	101	31.2
Occupational status		
Farmer	119	36.7
House wife	39	12.0
Employed	57	17.6
Merchant	32	9.9
Daily laborer	32	9.9
Other	45	13.9
Characteristics		Mean (± SD)
Median Age		39 (± 15) year

#### Clinical/surgical features of the patients

Majority of the patients 267 (82.4%) carried out emergency surgery. Majority 303 (93.5%) and 239 (73.8%) of patients have no co-morbidity and history of surgery respectively. Nine out of ten (n = 289) had less than 2 weeks duration of disease. More than one third of the participants 121 (37.3%) had disease of gastro intestinal system (Table 2).

**Table 2:** Clinical feature of admitted surgical patients Northern Ethiopia, 2016 (N = 324)

Characteristics	Frequency	Percentage
Duration of disease (week)		
≤ 2	289	89.2
> 2	35	10.8
History of surgery		
Yes	85	26.2
No	239	73.8
Type of surgery		
Elective	178	54.9
Acute/emergency	146	45.1
Co morbidity		
No	303	93.5
Yes	21	6.5
Type of disease		
Gastro intestinal system	121	37.3
Cardio vascular system	14	4.3
Urinary system	31	9.60
Muscular system	74	22.8
Skeletal system	73	22.5
Nervous system	11	3.4

#### Nutritional status of admitted surgical patients

Thirty one percent (n = 102) of patients were underweight and mean MUAC was 21.5 cm (± 2.7). Three out of 100 females 10 (3.1%) were at risk of under nutrition measured using waist circumference. About 71 (21.9%) were at risk of under nutrition estimated using waist hip ratio and 30 (9.3%) respondents had abnormal level of hemoglobin during admission. Sixty nine percent (n = 224) of the admitted patients had low diet diversity score 224 (69%) (Table 3).

**Table 3:** Nutritional status of admitted surgical patients, Northern Ethiopia, 2016 (N = 324)

Characteristics	Frequency	Percent
BMI		
Under weight	102	31.5
Normal weight	221	68.2
Over weight	1	0.3
Waist circumference		
Male (cm)		
< 94	208	64.2
≥ 94	2	0.6
Female (cm)		
< 80	104	32.1
≥ 80	10	3.1
Waist hip ratio		
High risk	71	21.9
Moderate risk	83	25.6
Low risk	170	52.4
Hemoglobin		
Abnormal ( < 11 g/dl)	30	9.3
Normal ( ≥ 11 g/dl)	294	90.7
IDDS		
High DDS	11	3.4
Medium DDS	89	27.4
Low DDS	224	69.2

Characteristics	Frequency	Mean (± SD)
MUAC	324	21.5 (± 2.7)cm
Hip circumference	324	52 (± 23)cm
Waist circumference	324	46 (± 21) cm

#### Predictors of hospital length of stay among admitted surgical patients

In bi-variable Cox proportional regression analysis age, educational status, marital status, family members, type of transportation, occupational status, duration of current disease, history of surgery, type of surgery, co morbidity, body mass index, hemoglobin, Mid Upper Arm Circumference, IDDS, dark green leafy vegetables were significant.

After adjustment, the independent significant predictors of length of hospital stay were: the hazard of hospital stay were 3.7 times lower among patients who had less than two weeks duration of disease than those greater than two weeks duration [AHR = 3.7, 95% CI (2.35–5.82)]. Hospital length of stay in admitted surgical patients who did not have history of surgery had 1.4 times less hazard of hospital stay than those who had previous surgery (AHR = 1.41, 95% CI 1.17–1.86). Patients admitted with normal weight were 1.3 times more likely to reside shorter in hospital than underweight patients (AHR = 1.38, 95% CI 1.28–1.51). Additionally based on MUAC measurement admitted surgical patients with normal nutrition had 1.3 times lower probability of hospital stay than the counterpart (AHR = 1.29, 95% CI 1.04–1.62). Patients feeding high DDS were 2.6 times more likely to stay lower in hospital than patients feeding lower DDS (AHR = 2.64, 95% CI 1.14–6.14) (Additional file 1: Table S1).

### Discussion

A shorter hospital stay will reduce the cost per discharge and shift care from inpatient to less expensive post-acute settings and effective reduction of a hospital’s average length of stay is not a question of simply discharging patients earlier. Instead, it is ensuring that patients recover more quickly and reach the point at which they are ready to leave the hospital sooner [1, 8, 13-16].

As the finding of this study, patients with normal BMI had a shorter length of Hospital stay than underweight patients. Similar Studies from Switzerland, Korea, Portugal, Israel, Brazil, and Egypt supports this study [13, 15, 17-20]. A study found that, one day difference in stay between the normal weight and underweight of BMI. Such a delay may have financial and psychological implications, and may expose patients to additional risk of acquiring nosocomial conditions [21]. And also treatment decisions for patients such as laparoscopic versus open surgery or conservative versus immediate operative management may differ depending on their weight. Furthermore, obesity is known to increase the risk of other conditions such as cardiovascular diseases or thrombo-embolic disease that may prolong hospitalization. But this study was in contrast to study done in United States [22] which observed that patients with higher BMI had shorter hospital stays compared with patients with normal BMI. This result was observed even after adjusting for number of co-morbidities. This suggests that when differences in co-morbid conditions are accounted for patients with higher BMI they are tended to be discharged sooner.

This study revealed that nutritional status of the patients during admission measured using MUAC showed that patients with normal nourished had higher hazard of discharge. MUAC has been proposed as an alternative index of nutritional status when the collection of height and weight measurements is difficult, including in emergencies such as famines or refugee crises. This result is in line with studies done in Brazil, Portugal, India, and Vietnam which found an increase in length of stay in malnourished patients [12, 18, 23, 24].

Other predictor of length of stay was duration of the disease; patients with greater than two weeks of disease duration had higher length of hospital stay. This result is consistent with study done in India [25]. This indicates that duration of disease increases loss of appetite, complication, lower immunity and all this might lead to an increased hospital stay.

This study revealed that length of hospital stay was higher in those patients who had history of surgery. This result was in line with studies done in Tabriz Shahid Madani Cardiovascular Hospital [26], Iran [27]. This could be due to history of surgery might affect the current disease recovery rate and could lead to low immunity.

Another predictor of hospital length of stay was the IDDS revealed that patients with high DDS had lower hospital stay than patients feeding low DDS. Similar study done in Israel supports this [15]. Dietary diversity is remarkably associated with nutrient adequacy and represents diet quality. Diets with low diversity could increase the risk of nutrient deficiencies especially in vulnerable populations such as diseased individuals, elderly and women [28]. Individuals with high diet diversity might develop immunity and can reduce infection, complications.

### Conclusion

Independent predictors of hospital length of stay in admitted surgical patients were found to be duration of disease, history of surgery, nutritional status (BMI, MUAC). Therefore it is recommended that patients at the time of hospital admission need to be screened for malnutrition, dietary supplementations in hospitals should be based on their nutritional status and specific deficiencies should be evaluated in patients found to be malnourished at the time of hospital admission. Researchers are needed to examine or give attention the occurrence of malnutrition upon hospital-admission and hospital-acquired malnutrition among all patients admitted to Hospital.

## Limitation of the study

This study doesn’t consider the effect of hospital acquired malnutrition during stay; there might be social acceptability bias and early discharge due to shortage of bed in the hospital. The sample size is small in contrast to the variables so needs careful interpretation of the key findings.

## Additional file

**Additional file 1: Table S1.** Bi and multivariable analysis predictors of length of hospital stay, Northern Ethiopia 2016 (N = 324).

## Data Availability

The datasets used and/or analyzed during the current study is available from the corresponding author on request.

## Abbreviations

BMI: body mass index
IDDS: individual diet diversity score
IDF: international diabetic foundation
LOS: length of stay
MUAC: mid upper arm circumference
WC: waist circumference
WHO: World Health Organization
WHR: waist hip ratio

## References

[1] Ilesanmi OS, Fatiregun AA (2014). Length of stay of surgical inpatients at University College Hospital, Ibadan, Nigeria. Br J Med Med Res..

[2] Laky B, Janda M, Kondalsamy-Chennakesavan S, Cleghorn G, Obermair A (2010). Malnutrition and quality of life association with prolonged length of hospital stay among patients with gynecological cancer in Australia. BioMed Centeral..

[3] Sang SLW, Chaturvedi R, Alam A, Samoukovic G, Varennes B, Lachapelle K (2013). Preoperative hospital length of stay as a modifiable risk factor for mediastinitis after cardiac surgery in Canada. J Cardiothor Surg..

[4] Group DMS (2015). Recognition and treatment of under nutrition in hospital inpatients and outpatients.

[5] Shpata V, Prendushi X, Kreka M, Kola I, Kurti F (2014). Malnutrition at the time of surgery affects negatively the clinical outcome of critically ill patients with gastrointestinal cancer in University of Hospital of Albania..

[6] Goiburu ME, Goiburu MMJ, Bianco H, Díaz JR, Alderete F (2006). The impact of malnutrition on morbidity, mortality and length of hospital stay in trauma patients in Brazil. Nutr Hosp..

[7] Leandro-Merhi VA, Aquino J, Chagas JFS (2010). Nutrition status and risk factors associated with length of hospital stay for surgical patients school of medicine, Puc-Campinas-SP, Brazil. J Parent Enteral Nutr..

[8] Saunders J, Smith T (2010). Malnutrition: causes and consequences Southampton University Hospitals Southampton. CME Nutrition..

[9] Adugna A. lesson 13 Health Institutions and Services.

[10] Smith PE, Smith AE (1997). High-quality nutritional interventions reduce costs. Financ Manage..

[11] Barker LA, Gout BS, Crowe TC (2011). Hospital malnutrition: prevalence, identification and impact on patients and the healthcare system in Victoria, Austria. Int J Environ Res Public Health..

[12] Silva HGV, Santos NOS, Ribeiro LLJ, Moreira ASB (2012). Nutritional assessment associated with length of inpatients’ hospital stay Rio de Janeiro State University Brazil. Nutr Hosp..

[13] Pichard C, Kyle UG, Morabia A (2004). Nutritional assessment: lean body mass depletion at hospital admission is associated with an increased length of stay in USA. Am J Clin Nutr.

[14] Dickaut S, DeLee J, Page C (1984). Nutrition status: importance in predicting wound healing after amputation. J Bone Joint Surg..

[15] Luiz A, Wick EC (2013). Risk factors for prolonged length of stay after colorectal surgery in Israel. J Coloproctol..

[16] OECD (2015). Average length of stay in hospitals in Health at a Glance 2015 OECD Indicators.

[17] Lee H-K, Choi H-S, Son E-J, Lyu E-S (2013). Analysis of the prevalence and risk factors of malnutrition among hospitalized patients in Busan Korea. Prev Nutr Food Sci..

[18] Correiamit D, Caiaffa WT, Silva ALD, Waitzberg DL (2001). Risk factors for malnutrition in patients undergoing gastroenterological and hernia surgery in University of São Paulo Medical School, Brazil: an analysis of 374 patients. Nutr Hosp..

[19] Leandro-Merhi VA, Aquino Jl, Camaro JG, Frenhani PB, Bernardi JLD, McClellan KCP. Clinical and nutritional status of surgical patients with and without malignant diseases in Brazil: cross-sectional study. Arq Gastroenterol. 2011; 48:1. 10.1590/s0004-28032011000100012 21537544

[20] Hai RA, Bakr Y (2008). nutritional status of hospitalized patients and its impact on morbidity, mortality and length of stay in Cairo. Bulletin of High Institute of Public Health..

[21] Zizza C, Herring AH, Stevens J, Popkin BM (2004). Length of hospital stays among obese individuals. Am J Public Health..

[22] Akinyemiju T, Meng Q, Vin-Raviv N (2016). Association between body mass index and in-hospital outcomes. Medicine..

[23] Haile A, Hailu M, Tesfaye E (2015). Prevalence and associated factors of malnutrition among adult hospitalized patients at Amhara National Regional State Referral Hospitals Ethiopia. Integrative Obesity and Diabetes..

[24] Young LS, Huong PTT, Lam NT, Thu NN, RD HTV, Hanh NL, et al. Nutritional status and feeding practices in gastrointestinal surgery patients at Bach Mai Hospital, Hanoi, Vietnam. 2015 10.6133/apjcn.092015.15 PMC5318161 27440685

[25] Mahakalkar CC, Modi S, Yeola M, Kaple MN, Patwardhan MA (2013). Malnutrition in hospitalized patients: a real concern in surgical outcomes Maharashtra India. Int J Res Med Sci..

[26] Gholivahidi R, Kooshavar H, Khodayari R (2006). The Study of patient’s length of stay and its associated factors in Tabriz Shahid Madani Cardiovascular Hospital. J Health Adminis..

[27] Khosravizadeh O, Vatankhah S, Bastani P, Kalhor R, Alirezaei S, Doosty F (2016). Factors affecting length of stay in teaching hospitals of a middle-income country. Electron Phys..

[28] Nachvak SM, Abdollahzad H, Mostafai R, Moradi S, Pasdar Y, Rezaei M (2017). Dietary diversity score and its related factors among employees of Kermanshah University of Medical Sciences. Clin Nutr Res..

