# Supplementary material for: Nutritional status significantly affects hospital length of stay among surgical patients in public hospitals of Northern Ethiopia: single cohort study
Source: BMC Res Notes. 2019 Jul 15;12:416. doi: 10.1186/s13104-019-4451-5 (PMC6631984; doi:10.1186/s13104-019-4451-5)
Supplement: Supplementary file 1 — Additional file 1: Table S1. Bi and multivariable analysis predictors of length of hospital stay, Northern Ethiopia 2016 (N = 324). [file 13104_2019_4451_MOESM1_ESM.docx]

**Table S1: Bi and multivariable analysis predictors of length of hospital stay, Northern Ethiopia 2016 (N = 324)**

| Characteristics | Number at risk (%) | Event  (Discharged) | HR 95%CI | |
| --- | --- | --- | --- | --- |
|  |  |  | **Crude** | **Adjusted** |
|  |  |  |  |  |
| Age of participants |  |  |  |  |
| 18-40 | 201(62) | 201 | 1 | 1 |
| 41-64 | 97(29.9) | 97 | 0.720(0.561-0.925) | 0.567(0.094-1.470)* |
| >64 | 26(8.1) | 26 | 0.603(0.399-0.910) | 0.693(0.045-1.744)* |
| Educational status |  |  |  |  |
| No formal education | 137(42.3) | 137 | 1 | 1 |
| Grade 1-4 | 22(6.8) | 22 | 0.834(0.530-1.311) | 0.705(0.420-1.185) |
| Grade 5-8 | 35(10.8) | 35 | 1.368(0.942-1.986) | 1.426(0.910-2.234) |
| Grade 9-10 | 49(15.1) | 49 | 1.267(0.912-1.760) | 0.929(0.609-1.418) |
| Grade 11-12 | 29(9.0) | 29 | 1.727(1.152-2.588) | 0.852(0.486-1.494) |
| College and above | 52(16.0) | 52 | 1.098(0.795-1.517) | 1.449(0.810-2.590) |
| Transportation Type |  |  |  |  |
| Foot | 31(9.6) | 31 | 1 | 1 |
| private care | 11(3.4) | 11 | 1.670(1.115-2.503) | 0.972(0.324-2.915) |
| public care | 169(52.2) | 169 | 0.903(0.367-2.222) | 0.910(0.544-1.522) |
| Government car | 12(3.7) | 12 | 1.089(0.852-1.393) | 0.948(0.428-2.102) |
| Ambulance | 101(31.2) | 101 | 1.245(0.682-2.274) | 0.827(0.488-1.401) |
| Duration of disease |  |  |  |  |
| ≤ 2 weeks | 289(89.2) | 289 | 2.608(1.762-3.856) | **3.70(2.352-5.820)***** |
| >2 week | 35(10.8) | 35 | 1 | 1 |
| History of surgery |  |  |  |  |
| Yes | 85(26.2) | 85 | 1 | 1 |
| No | 239 (73.8) | 239 | 1.309(1.020-1.680) | **1.409(1.169-1.858)*** |
| Type of surgery |  |  |  |  |
| Elective | 143(44.1) | 143 | 1 | 1 |
| Acute/Emergency | 181(55.9) | 181 | 1.520(1.210-1.910) | 1.234(0.954-1.594) |
| Co morbidity |  |  |  |  |
| No | 303(93.5) | 303 | 1.537(0.986-2.395) | 1.361(0.825-2.247) |
| Yes | 21(6.5) | 21 | 1 | 1 |
| Body Mass Index |  |  |  |  |
| Under weight | 102(31.5) | 102 | 1 | 1 |
| Normal weight | 222(68.5) | 222 | 2.439(1.860-3.200) | **1.38(1.282-1.513)***** |
| Hemoglobin |  |  |  |  |
| Abnormal | 30(9.3) | 30 | 1 | 1 |
| Normal | 294(90.7) | 249 | 1.744(1.193-2.549) | 1.310(0.865-1.985) |
| MUAC |  |  |  |  |
| Under nutrition | 157(48.5) | 157 | 1 | 1 |
| Normal nutrition | 167(51.5) | 167 | 1.296 (1.036-1.616 | **1.296 (1.036-1.616)*** |
| IDDS |  |  |  |  |
| High DDS | 11(3.4) | 11 | 2.670(1.438-4.957) | **2.644(1.138-6.143)*** |
| Medium DDS | 89(27.4) | 89 | 1.216(0.948-1.560) | 1.175(0.901-1.533) |
| Low DDS | 224(69.2) | 224 | 1 | 1 |

NB. *=P-Value <0.05, **= P-Value ≤0.01 and ***=P-Value ≤0.001
